# Supplementary material for: Learning curve and surgical outcome of robotic assisted colorectal surgery with ERAS program
Source: Sci Rep. 2022 Nov 29;12:20566. doi: 10.1038/s41598-022-24665-w (PMC9709162; doi:10.1038/s41598-022-24665-w)
Supplement: Supplementary file 1 — Supplementary Information 1. [file 41598_2022_24665_MOESM1_ESM.zip › LSC Raw data-Ñ[▒K/123/O_ñw╝╨░O▒Kñσ.pdf]

正式摘要(電子簽章：000000215575856)

請選擇...reporter

臺中榮民總醫院

Operation Note

[redacted]

- 手術日期：2021/03/24
- 手術醫師：林俊余，CR 林陳彥，R 楊立宇
- Anesthesia : GE
- Position : Lithotomy
- Aseptic agents : Betadine Aqua , Zephnan

Diagnosis

- Pre-operative diagnosis : Rectal colon tumor s/p loop S-colostomy
- Post-operative diagnosis : s/p conventional low anterior resection + loop ileostomy formation

Surgical Indication

- For removing the primary tumor

Operation

- 1. Conventional low anterior resection with loop ileostomy (Initially laparoscopic was applied)
  2. Intraoperative colonoscopy : (-)
  3. High ligation (+) : At IMA root. IMV and left colic artery were also ligated.
  4. EndoGIA (60-4.1) X2 were applied to resect the rectum
  5. Proximal cut end: 10 cm, distal cut end: 3 cm
  6. Loop ileostomy was created and fixed over LLQ of abdomen (About 20 cm prior to cecum). (The previous colostomy wound)
  7. One 12 Fr. penrose drainge tube was placed from LLQ of abdomen and inserted into the pelvic cavity
  8. The colon was taken down from the splenic flexure site
  9. Hyalobarrier gel was applied below the central extended wound

Finding

- 1. A ulcerative tumor located at rectum area
  2. No obvious other tumor could be palpated over the D- and S-colon
  3. No obvious ascites was found
  4. Moderately adhesion was presented over the S-colon arae ; But the rectum was severely adhered to the nearby tissue over the pelvic area
  5. Bilateral doughnuts : intact ; Air leak test: Done and no air leak was found

Procedure

- 1. General endotracheal anesthesia was induced and then the patient was placed in modified Trendelenburg lithotomy position. Preoperative antibiotics were given. A Foley catheter and a nasogastric tube were placed. The abdomen was prepared and draped in standard sterile fashion.
  2. Initially, the loop S-colostomy was taken down and temporarily closed with continuous running suture via 2-O mersilk.
  3. A small vertical incision was made right next to the umbilicus and a 12 mm trocar was inserted into the peritoneal cavity by open method. Peumoperitoneum was established with CO2 inflation. Laparoscopy was inserted and then another 12 mm trocar was inserted over RLQ. Extra two 5 mm trochars were inserted over RUQ and LUQ. The operative findings were mentioned as above.
  4. The small bowel was inspected and retracted to the right by grasper. The sigmoid colon was then held by grasper and the peritoneum overlying the mesentery was then scored with electrocautery. The inferior mesentery artery was identified, ligated below the left colic artery with endoclips, and

- transected, so was inferior mesentery vein. Dissection was done above the Gerota's fascia and toward the peritoneal attachments.
  5. The arcade vessels to the sigmoid colon were also divided. Distal descending colon and upper rectum were mobilized.
  6. The colon was mobilized from retroperitoneum overlying the sacral promontory and into the pelvis. The incision was extended laterally and medially across the peritoneal reflection.
  7. Due to severely adhesion was noted, we switched our procedure to open method. The central laparoscopic-trochar wound was further extended above and below the umbilicus area. Rogan was then set up.
  8. The mesorectum was then mobilized using sharp dissection over the level of pelvic floor muscle. Then distal descending colon and rectum were mobilized as possible.
  9. Totally two linear staplers (EndoGIA 60-4.1) were used to divide the lower rectum.
  10. The avascular line of Toldt along the left paracolic gutter was incised from the D-colon toward the splenic flexure as possible.
  11. The lesser sac was approached and the omentum was resected carefully. The gastrocolic omentum was divided as far to the left as possible until the splenic flexure was no longer tethered.
  10. After the splenic flexure had been taken down, the proximal colon was then taken out for carefully inspection, and to make sure it has an excellent blood supply. The mesenteric fat was gently cleared from the terminal 2-3 mm of the bowel ends.
  12. The field was isolated with moist pad and the bowel opened and sized. It was determined that a 29 mm circular stapler could be accommodated. A pursestring suture of 2-0 prolene was placed, the anvil was inserted, and then the pursestring suture was tied.
  13. The anal canal was irrigated with distil water and then the circular stapler was introduced through the anus and pierced through the mid-portion of the stapler line.
  14. The proximal bowel with anvil in place was engaged with the circular stapler and the stapler was then fired. Two complete donuts were obtained and the distal one was submitted to pathology for margin confirmation.
  15. The anastomosis was tested for leaks with irrigation solution and air introduced through the rectum and was found to be patent and intact.
  16. The possible bleeder was then checked thoroughly.
  17. The previous colostomy wound was then selected as ileostomy site.
  18. The desired terminal ileum (20 cm prior to the cecum) was selected. A window was created under the ileum by dividing the mesentery adjacent to the bowel wall with electrical cautery carefully. Then it was looped via Silicone Vessel loops.
  19. The wound over the LUQ trochar site was then served as drainage tube insertion site. The tube was inserted over pelvic area.
  20. The terminal ileum was then dragged out from the ostomy site carefully and a plastic bridge (Rod) was placed via the window.
  21. Hyalobarrier gel was applied below the central extended wound
  22. The fascia of midline wound was closed with interrupted sutures via Num. 1 Vicryl. The trochar wound was further closed with interrupted method via 1-O Vicryl.
  23. After irrigation the wound with N/S, the skin was closed with interrupted 3-0 Nylon
  24. The loop ileostomy was matured with multiple interrupted mucocutaneous suture via 4-0 Monocryl.
  25. The loop ileostomy was well formed and viable. An ostomy bag was placed.
  26. The patient tolerated the procedure well and was brought to the POR in stable condition
- Estimated blood loss: 200 c.c
- 器械數量: 正確
- 紗布數量: 正確

Image

- PAINT
  - abdomen

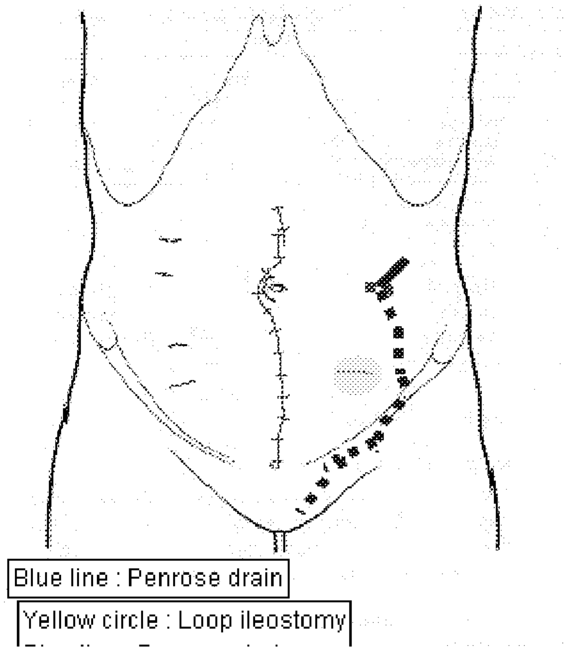

- colon & rectum

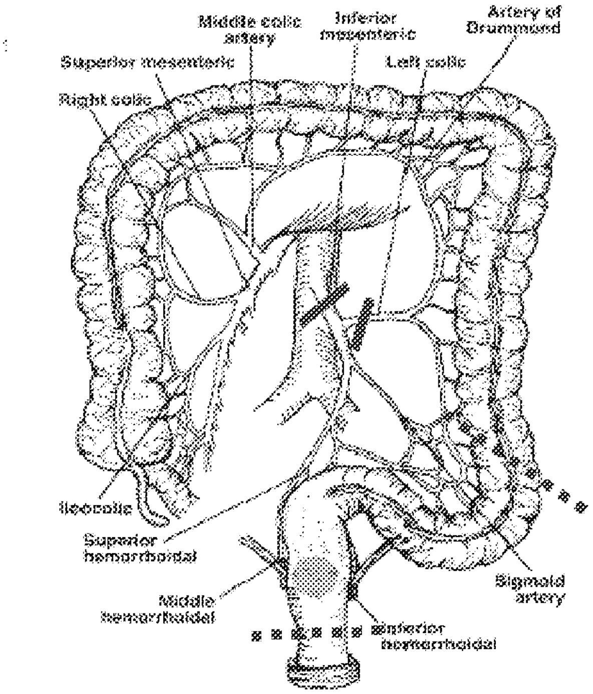

- The R-S colon

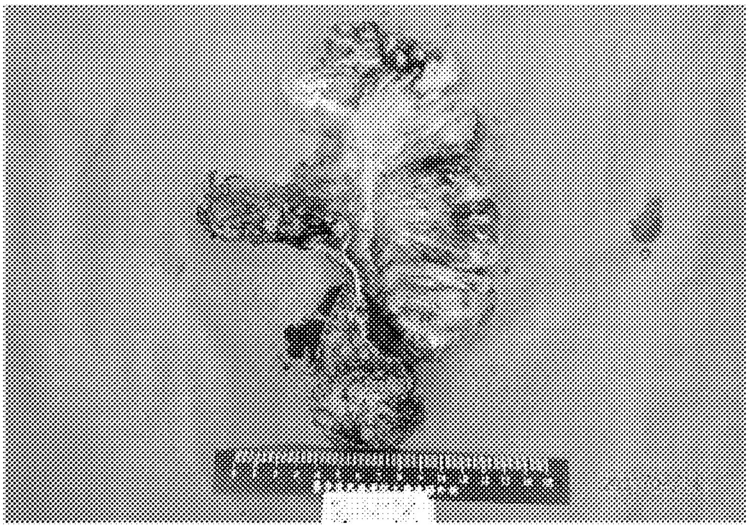

The tumor could be clearly seen

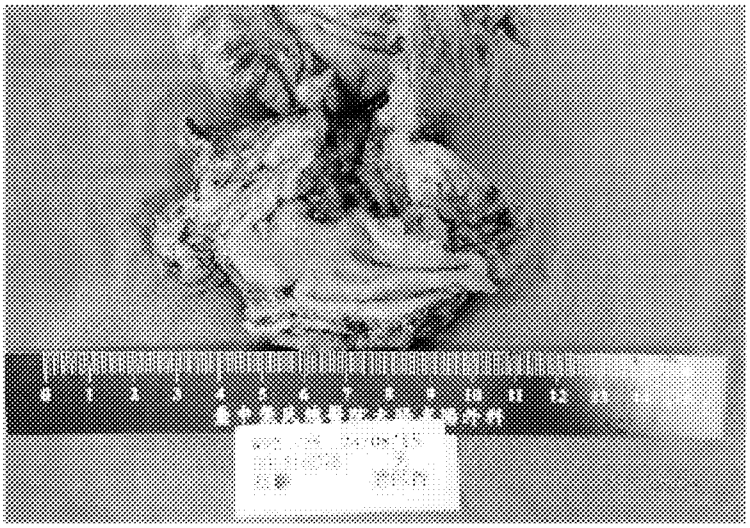

主治 2222H林俊余 / 醫師 2301H林陳彥

正式摘要(電子簽章：**000000215575856)**

請選擇...reporter

doctorRank:B
